# MAC/MAB–RCS: An Integrative Regulatory Control Framework for Risk Stratification and Personalized Intervention in Addiction Psychiatry

**DOI:** 10.3390/brainsci16020187

**Published:** 2026-02-03

**Authors:** Anna Makarewicz, Remigiusz Recław, Anna Grzywacz, Jolanta Chmielowiec, Krzysztof Chmielowiec

**Affiliations:** 1 Department of Hygiene and Epidemiology, Collegium Medicum, University of Zielona Góra, 28 Zyty St., 65-046 Zielona Góra, Poland; makarewicz81@gmail.com (A.M.); chmiele@vp.pl (K.C.); 2 Independent Laboratory of Behavioral Genetics and Epigenetics, Pomeranian Medical University in Szczecin, Powstancow Wielkopolskich 72 St., 70-111 Szczecin, Poland; grzywacz.anna.m@gmail.com; 3 Department of Medical Sciences and Public Health, Gdansk University of Physical Education and Sport, Kazimierza Górskiego 1 St., 80-336 Gdansk, Poland; 4 Department of Nursing, Collegium Medicum, University of Zielona Góra, 28 Zyty St., 65-046 Zielona Góra, Poland; j.chmielowiec@inz.uz.zgora.pl

**Keywords:** substance-related disorders, craving, executive function, psychological stress, precision medicine, neurobiology, impulse control disorders

## Abstract

Objectives: Addiction disorders remain a major challenge in contemporary psychiatry due to high relapse rates and significant individual and societal burden. Despite advances in addiction neurobiology, current diagnostic frameworks and dominant models offer limited tools for early risk identification and dynamic support of clinical decision-making across the course of treatment. The aim of this narrative review is to introduce the MAC/MAB–RCS model as an integrated conceptual framework for risk stratification and personalized intervention in addiction psychiatry. Methods: The proposed model integrates evidence from four complementary domains: genetic, epigenetic, and stress-axis biomarkers; functional brain network organization; and psychological/psychiatric dimensions relevant to addictive behaviors. These domains are synthesized into a unified conceptual structure designed to capture dynamic regulatory processes underlying addiction vulnerability. Results: At the core of the model lies the Regulatory Control State (RCS), a latent higher-order construct representing an individual’s dynamic regulatory capacity through the integration of cognitive control, emotional regulation, and motivational drive modulation. Disruption of the RCS is conceptualized as a shared transdiagnostic mechanism driving craving escalation, compulsive behavior, and relapse vulnerability, independent of substance class or specific addictive behavior. Conclusions: The MAC/MAB–RCS model aligns with the principles of precision psychiatry by offering a pragmatic, clinically oriented translational framework with potential applicability across clinical settings, bridging neurobiological research and clinical practice. The review discusses its relationship to existing models, potential clinical and systemic applications, key limitations, and priorities for future validation studies.

## 1. Introduction

Addiction disorders are widely conceptualized as chronic, relapsing conditions with substantial individual and societal burden, underpinned by dysregulation across cognitive control, affective, motivational, and stress-related systems [1,2]. At the same time, the field continues to refine how “brain-based” models should be interpreted in clinically responsible and non-reductionist ways [1].

Despite major advances in addiction neuroscience, current classification systems (DSM-5-TR; ICD-11) remain predominantly descriptive and tend to capture symptom patterns that become most evident at later stages of illness [3,4]. This limits their utility for early risk identification, dynamic monitoring of relapse vulnerability, and guiding treatment intensity and personalization in routine care [5].

In response, precision psychiatry aims to integrate biological and clinical data to support risk stratification and intervention selection [6]. In addiction psychiatry, this shift highlights the need for functional, state-sensitive models that can detect early regulatory destabilization and characterize risk trajectories over time, beyond static diagnostic categories [7].

However, existing frameworks (e.g., RDoC, ANA, and neurocircuitry models) often provide domain-based descriptions without specifying a single clinically interpretable state-level construct that integrates multidomain signals into actionable clinical decisions. To address this gap, we propose the MAC/MAB–RCS model, centered on the Regulatory Control State (RCS), an integrative state-level construct reflecting momentary self-regulatory capacity. We then position the framework relative to established models and outline its potential clinical applications, limitations, and priorities for validation research. This gap is addressed through the proposed Regulatory Control State (RCS), detailed in Section 3.2.

Importantly, the proposed MAC/MAB–RCS model does not aim to replace or compete with existing frameworks such as the Research Domain Criteria (RDoC). While RDoC includes state-sensitive constructs (e.g., acute threat, frustrative nonreward), these constructs are organized within parallel functional domains and are primarily intended to support research-oriented classification rather than real-time clinical decision-making. In contrast, the Regulatory Control State (RCS) is conceptualized as a higher-order, integrative state-level construct that synthesizes multidomain information into a single, clinically interpretable reference point.

Rather than representing a specific affective or motivational state, RCS reflects momentary stability of self-regulatory capacity as an emergent property of interacting cognitive control, emotional regulation, and motivational drive systems. This integrative function distinguishes RCS from domain-specific state constructs by explicitly linking state-level assessment to proportional intervention selection and sequencing. In this sense, the novelty of the MAC/MAB–RCS framework lies not in the identification of new state phenomena, but in the formulation of a unifying clinical state parameter intended to translate multidimensional neurobiological and psychological signals into actionable, time-sensitive treatment strategies.

## 2. Materials and Methods

### 2.1. Design of the Review and Conceptual Framework Development

This work is a narrative review incorporating elements of conceptual synthesis, aimed at developing a theoretical framework to facilitate the translation of neurobiological knowledge into clinical practice in addiction psychiatry. The article does not present original empirical data; instead, it focuses on a critical analysis of existing models and the identification of conceptual gaps that limit their clinical applicability.

The development of the MAC/MAB–RCS model involved: (1) an examination of current theoretical paradigms in addiction research, (2) the identification of shared regulatory mechanisms associated with relapse and loss of control, and (3) the synthesis of these mechanisms into a coherent, functionally oriented clinical construct.

### 2.2. Literature Search Strategy and Scope

The literature review covered publications from 2022 to 2025 identified through searches of PubMed (National Library of Medicine, Bethesda, MD, USA), Web of Science (Clarivate, Philadelphia, PA, USA), and Scopus (Elsevier, Amsterdam, The Netherlands). The search strategy included combinations of terms related to addiction disorders, cognitive control, craving, stress, emotional regulation, impulsivity, compulsivity, functional brain networks, and precision psychiatry. The selected time window was intended to capture the most recent conceptual and translational developments in addiction neuroscience and precision psychiatry, reflecting rapid advances in neurobiological models, network-based approaches, and state-sensitive conceptual frameworks. Earlier foundational models were selectively included when necessary to contextualize contemporary developments.

Particular emphasis was placed on review articles, conceptual papers, and translational studies with high clinical relevance, including frameworks such as the Research Domain Criteria (RDoC), the Addictions Neuroclinical Assessment (ANA), and contemporary models of addiction neurocircuitry. We prioritized English-language reviews, conceptual papers, meta-analyses, and clinically oriented translational studies relevant to relapse risk dynamics and regulatory control processes. Reference lists of key articles were manually screened to identify additional relevant publications. The review was not intended to be exhaustive but to capture conceptually and clinically relevant developments informing regulatory control mechanisms in addiction.

### 2.3. Analytical Framework and Criteria for Synthesis

The literature analysis was qualitative in nature and focused on identifying shared elements across theoretical models, particularly within domains related to executive control, reward processing, stress reactivity, and emotional regulation. Competing theoretical constructs (e.g., self-regulation, inhibitory control, allostatic load) were evaluated based on their capacity to integrate multidomain evidence and their direct applicability to dynamic clinical decision-making. Concepts that primarily described isolated functional domains or static vulnerability traits were not adopted as organizing constructs but were instead incorporated as contributory components within the broader integrative framework.

Based on this qualitative synthesis, we observed a persistent translational gap between rapidly expanding neurobiological evidence and the availability of a clinically actionable, higher-order parameter that can integrate these data to support therapeutic decision-making.

Although the RCS model is grounded in the synthesis of narrative reviews and meta-analytic literature (e.g., meta-analyses addressing emotional dysregulation [8] or relapse prevention strategies [9]), these sources inherently entail methodological limitations. Such limitations include heterogeneity across included studies, variability in outcome definitions, and the relative scarcity of randomized controlled trial data directly informing integrative regulatory models. For example, the meta-analysis by Tabugan et al. [9] is primarily narrative in nature, which may introduce selection bias and limit its capacity to provide empirical validation of specific intervention effects. A critical appraisal of these sources underscores the need to integrate conceptual synthesis with future prospective and longitudinal data, to avoid oversimplification when formulating claims regarding shared transdiagnostic regulatory mechanisms.

Given the narrative and conceptual nature of the present review, individual studies were not weighted using formal quantitative quality scores. Instead, prioritization during conceptual synthesis was guided by convergence across independent lines of evidence, conceptual relevance to state-level regulatory processes, and translational applicability to clinical decision-making. Greater interpretative weight was assigned to findings that demonstrated consistency across multiple study designs, populations, or methodological approaches, rather than to isolated or single-study effects. To further minimize selection bias, emphasis was placed on integrative reviews, meta-analyses, and translational frameworks that synthesized evidence across domains, while individual empirical studies were primarily used to illustrate convergent mechanisms rather than to support standalone claims. This approach was intended to balance conceptual depth with methodological transparency while remaining appropriate for a narrative review. To further mitigate selection bias, findings were cross-referenced with available meta-analyses and integrative reviews where possible (e.g., González-Roz et al. [8] on emotional dysregulation).

### 2.4. Development of the Regulatory Control State (RCS) Construct

The Regulatory Control State (RCS) construct was developed in response to this identified conceptual gap as an integrative, state-level organizing concept for the synthesis presented in this review.

In contrast to models based on relatively stable traits or parallel assessment domains, RCS was designed as a higher-order functional indicator, enabling the interpretation of multilevel data in the context of real-time clinical decision-making. In future validation work, RCS can be operationalized as a composite, state-sensitive index informed by repeated clinical/EMA measures (e.g., craving, stress load, affect regulation), cognitive control indicators, and selected stress-axis and biological markers, with predictive validity tested longitudinally.

### 2.5. Ethical Considerations and Scope of Application

The MAC/MAB–RCS model constitutes a conceptual framework rather than a deterministic diagnostic or predictive tool. Biological, neurobiological, and psychological data are interpreted as indicators of vulnerability and regulatory dynamics, not as bases for rigid patient categorization or definitive clinical prognostication.

The application of the model is limited to supporting clinical reasoning, structuring the interpretation of multilevel data, and informing the design of validation studies. This approach is consistent with the ethical principles of precision psychiatry and aims to minimize the risks of biological overinterpretation and clinical reductionism. The framework also acknowledges risks related to stigma, inequitable access to biomarker assessments, and population-level confounding; therefore, any future implementation should follow principles of proportionality, transparency, and fairness in line with responsible precision psychiatry.

## 3. Results: Conceptual Framework of the MAC/MAB–RCS Model

### 3.1. Identification of a Core Regulatory Dimension in Addiction

An analysis of contemporary neurobiological and clinical models of addiction reveals a common and recurring mechanism underlying symptom escalation and relapse vulnerability: the progressive destabilization of an individual’s regulatory capacities. Regardless of substance type or the specific form of addictive behavior, loss of control manifests as a disruption in the integration of cognitive, emotional, and motivational processes, leading to the dominance of reactivity over control [10,11].

Existing theoretical frameworks—including addiction neurocircuitry models, transdiagnostic approaches, and domain-based research systems—describe these processes at the level of neural networks or psychopathological dimensions. However, they less frequently provide a higher-order, functional indicator capable of capturing the patient’s current regulatory state in a clinically actionable manner [8,12,13].

### 3.2. Regulatory Control State (RCS) as a Functional Clinical Construct

Based on the literature analysis, the Regulatory Control State (RCS) construct was identified as a higher-order functional indicator integrating key regulatory mechanisms of clinical relevance in addiction disorders. RCS is defined as a dynamic state of self-regulatory capacity arising from the integration of three core components: cognitive control, emotional regulation, and motivational drive modulation [14,15].

RCS does not correspond to a single biological marker or an isolated psychological trait. Instead, it reflects the current stability of regulatory systems as an integrated whole. RCS destabilization is proposed as an upstream process that may precede clinically observable relapse, consistent with evidence on dynamic relapse vulnerability [16]. Unlike domain-specific state constructs commonly described in research frameworks, RCS is explicitly formulated as a clinician-facing construct intended to support dynamic treatment calibration rather than domain-level symptom characterization. In contrast to constructs such as self-regulation, which typically emphasize domain-specific control processes, or allostatic load, which reflects cumulative physiological burden, RCS is formulated as a higher-order, state-level construct capturing the momentary stability of interacting regulatory systems. Crucially, RCS is explicitly linked to proportional intervention sequencing rather than descriptive characterization alone.

Clinically, RCS may be approximated using state-level indicators such as craving volatility/cue reactivity, stress load with affective lability, and real-world executive control failures (e.g., inhibition lapses, impulsive choices), complemented by early signs of compulsive routine reactivation. These anchors are intended to guide operationalization in validation studies rather than to define a fixed checklist.

### 3.3. Structure of the MAC/MAB–RCS Model

The MAC/MAB–RCS model was designed as a clinical framework in which the Regulatory Control State (RCS) serves as the overarching parameter guiding therapeutic decision-making. The stability or destabilization of RCS determines both the intensity of intervention and the selection of the dominant therapeutic strategy.

The architecture of the model is based on two complementary clinical strategies:

MAC (Minimizing Addiction Craving), which targets the reduction in craving as an early and sensitive indicator of regulatory destabilization and a predictor of relapse [17,18], and MAB (Modulation of Addictive Behaviors), which focuses on modifying compulsive behaviors and strengthening adaptive regulatory patterns that support the restoration of executive control [19].

**Figure 1:**
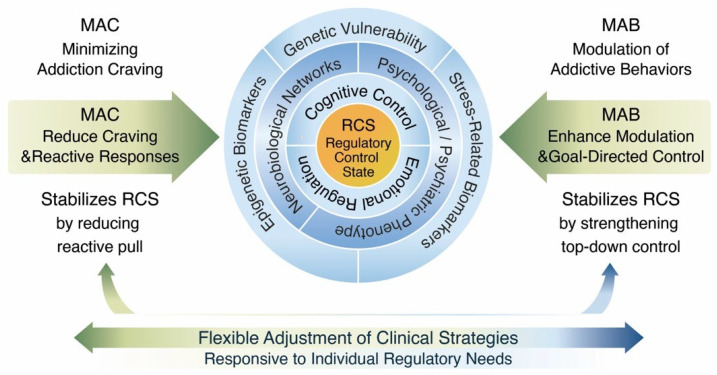
Conceptual structure of the MAC/MAB–RCS model.

MAC and MAB do not function as discrete treatment protocols but rather as flexible interventional components. Their relative proportion and intensity are dynamically adjusted according to the patient’s current RCS. The conceptual architecture of the MAC/MAB–RCS model is illustrated in Figure 1, highlighting the central role of regulatory control stability in guiding proportional clinical strategies.

Importantly, MAC and MAB are not tied to specific schools of therapy; rather, they represent proportional functional targets—reactivity/craving versus goal-directed control and behavioral modulation—that can be implemented via pharmacological, psychotherapeutic, behavioral, and digital intervention components.

The Regulatory Control State (RCS) represents a central, dynamic functional construct integrating cognitive control, emotional regulation, and motivational drive. Multidomain inputs—including genetic vulnerability, epigenetic and stress-related mechanisms, neurobiological network functioning, and psychological phenotype—influence the current stability of RCS. The model illustrates how MAC and MAB strategies are flexibly and proportionally adjusted to the individual’s regulatory control state, supporting adaptive and personalized clinical decision-making.

This conceptual structure provides the foundation for a dynamic interpretation of relapse risk, discussed in the following section.

Conceptually in practice, MAC-dominant periods are indicated by heightened craving volatility, stress-driven reactivity, and imminent loss-of-control risk signals, whereas MAB-dominant periods become feasible when craving burden is stabilized and cognitive control can be trained/leveraged to consolidate adaptive routines.

### 3.4. Dynamic Interpretation of Risk Within the MAC/MAB–RCS Framework

Within the MAC/MAB–RCS framework, relapse risk is interpreted dynamically, based on the assumption that the relative dominance of clinical strategies shifts as a function of the stability of the Regulatory Control State (RCS).

**Figure 2:**
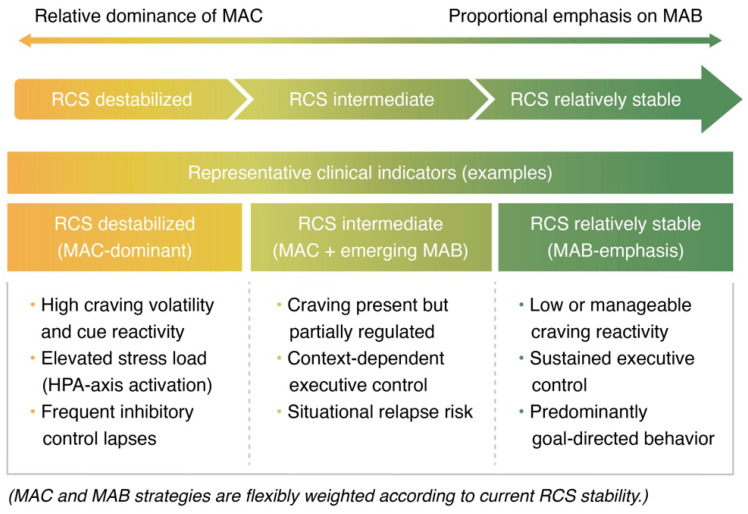
Functional relationship between Regulatory Control State (RCS) stability and clinical strategies within the MAC/MAB–RCS framework.

As illustrated in Figure 2, greater emphasis is placed on MAC strategies under conditions of RCS destabilization, focusing on the reduction in craving and reactive responding. As RCS stability increases, the relative importance of MAB strategies grows, supporting executive control and goal-directed behavioral regulation.

The schematic illustrates a proportional shift between Minimizing Addiction Craving (MAC) and Modulation of Addictive Behaviors (MAB) strategies as a function of current RCS stability. Lower RCS stability is associated with a greater emphasis on craving reduction and reactive response control (MAC), whereas increasing RCS stability corresponds to a stronger focus on executive control and goal-directed behavioral modulation (MAB).

The literature indicates that relapse risk in addiction disorders is dynamic, fluctuating in response to stress load, exposure to triggering cues, and changes in regulatory functioning [9,20]. Static approaches based solely on substance use history or diagnostic criteria fail to adequately capture this variability.

In the MAC/MAB–RCS model, relapse risk is conceptualized as a function of the current stability of the Regulatory Control State rather than as a fixed patient characteristic. This perspective enables a shift from reactive relapse management toward earlier identification and stabilization of regulatory dyscontrol before the emergence of a full symptomatic episode [21].

Accordingly, risk is framed as a trajectory of regulatory destabilization rather than a static probability estimate, supporting the concept of early-warning monitoring before overt relapse behaviors emerge.

The model yields testable hypotheses, including: (1) within-person decreases in RCS are expected to precede increases in craving variability and lapse risk; (2) MAC-weighted interventions will show larger short-term effects under low RCS, whereas MAB-weighted interventions will show larger consolidation effects under higher RCS; (3) multidomain vulnerability signals will moderate the threshold for RCS destabilization.

### 3.5. Positioning the Model Within Precision Psychiatry

The MAC/MAB–RCS model aligns with the paradigm of precision psychiatry by treating clinical heterogeneity as a central driver of treatment personalization. Rather than assuming uniform therapeutic pathways for specific diagnostic categories, the model supports the tailoring of interventional strategies to the patient’s functional regulatory profile [22].

Within this framework, the Regulatory Control State serves as an interface between neurobiological knowledge and clinical practice, enabling coherent interpretation of multilevel data in the context of individualized therapeutic decision-making.

Relative to RDoC, which organizes psychopathology into functional domains, and ANA, which structures multidomain assessment, the MAC/MAB–RCS framework proposes a state-level integrative construct (RCS) explicitly intended to scaffold time-sensitive clinical decisions. The model is compatible with stepped implementation: initial RCS estimation may rely on clinical and EMA-derived indicators, while biological and neuroimaging inputs can be incorporated where available to refine vulnerability stratification.

### 3.6. Prototype Clinical Operationalization of the Regulatory Control State

Although the Regulatory Control State (RCS) is not proposed as a formally validated metric, it can be approximated in routine clinical practice using a structured, heuristic assessment of state-level indicators that are already familiar to clinicians. The aim of this prototype operationalization is not precise quantification, but clinically meaningful estimation of current regulatory stability to guide proportional intervention selection.

At a practical level, RCS estimation may be informed by four core clinical dimensions: (1) craving intensity and volatility (e.g., cue reactivity, rapid escalation under stress), (2) stress load and affective lability, (3) observable executive control failures in daily life (e.g., impulsive decisions, inhibition lapses), and (4) early reactivation of compulsive behavioral routines. These indicators are interpreted jointly rather than independently, with emphasis on recent dynamics rather than static severity.

Within this framework, marked instability across multiple dimensions suggests a destabilized RCS and favors MAC-dominant strategies aimed at reducing craving and reactive responding. Conversely, relative stabilization of these indicators supports a shift toward MAB-oriented interventions focused on strengthening executive control, consolidating adaptive routines, and modifying maladaptive behavioral patterns. Importantly, this heuristic approach is intended to be feasible in low-resource settings and does not require biological or neuroimaging data to inform initial clinical decision-making.

Estimation of RCS in routine clinical practice does not rely on single symptoms or isolated observations, but on the convergence and temporal persistence of state-level indicators across sessions. Clinicians are guided by patterns of rapid and context-sensitive fluctuation, reduced stress tolerance, and repeated failures of executive control in real-world contexts. Transient elevations in stress or craving that resolve without loss of behavioral control are therefore not, by themselves, considered indicative of RCS destabilization. Rather, RCS destabilization is distinguished from adaptive or transient stress responses by the presence of functional spillover and sustained behavioral consequences. While normative stress reactions may involve elevated arousal or craving, they are typically followed by recovery of executive control and preservation of goal-directed behavior. In contrast, RCS destabilization is characterized by reduced recovery capacity, increasing cross-domain interference (e.g., stress amplifying craving and impairing inhibition), and a progressive erosion of behavioral control across contexts. This distinction supports pragmatic and reproducible clinical judgment without requiring access to advanced biomarkers or neuroimaging.

### 3.7. Illustrative Clinical Vignettes

Vignette 1 (MAC-dominant phase). A patient in early abstinence reports rapidly fluctuating craving triggered by minor stressors, accompanied by affective lability and repeated impulsive decisions despite strong motivation for change. Although insight is preserved, executive control repeatedly fails in real-world contexts. This pattern suggests a destabilized Regulatory Control State, indicating the need for MAC-dominant interventions focused on craving reduction, stress stabilization, and containment of reactive responses before attempting behavioral restructuring.

Vignette 2 (MAB-dominant phase). A patient with stabilized craving and reduced stress reactivity demonstrates improved consistency in daily routines and the ability to tolerate delayed rewards. While vulnerability remains under high-load conditions, executive control is sufficiently stable to support active modification of habitual behaviors. In this context, the Regulatory Control State is considered relatively stabilized, favoring a shift toward MAB-oriented strategies aimed at consolidating adaptive behaviors and strengthening goal-directed control.

These vignettes illustrate how RCS guides proportional shifts between MAC- and MAB-dominant strategies, emphasizing structured clinical judgment over rigid thresholds or algorithmic decision rules.

## 4. Multidomain Structure of the MAC/MAB–RCS Framework

### 4.1. Rationale for a Multidomain Approach

Available evidence indicates that single domains—genetic, neurobiological, or psychological—are insufficient to explain the dynamics of addiction disorders or to predict relapse risk at the individual level [23,24]. Addiction emerges as a phenomenon resulting from interactions across multiple levels of biological and clinical organization.

The MAC/MAB–RCS model adopts a multidomain approach in which information from different levels is not analyzed in isolation but interpreted through its impact on the stability of the Regulatory Control State. This perspective preserves data complexity while enhancing clinical interpretability.

Dynamic shifts between MAC and MAB strategies reflect the influence of multidomain factors on RCS stability, which are discussed in detail in the following subsections. Crucially, without a unifying state-level construct, multidomain data remain difficult to translate into time-sensitive clinical decisions regarding intervention intensity and sequencing.

### 4.2. Genetic Domain: Regulatory Vulnerability Stratification

Within the MAC/MAB–RCS model, the genetic domain serves to stratify regulatory vulnerability rather than to deterministically predict the development of addiction. Genetic variants related to reward system functioning, impulsivity, and stress regulation modulate the threshold for Regulatory Control State destabilization, increasing sensitivity to craving escalation and loss of control under environmental stressors [25,26].

From a clinical perspective, genetic information plays a contextual role, supporting risk assessment and decisions regarding monitoring intensity or intervention escalation, particularly in patients with a recurrent course of illness. The model explicitly rejects deterministic interpretations of genetic data, treating them as indicators of vulnerability rather than clinical destiny [27]. Within the MAC/MAB–RCS framework, genetic vulnerability is expected to lower the threshold for RCS destabilization, thereby increasing the relative clinical importance of MAC-oriented stabilization strategies under stress.

### 4.3. Epigenetic and Stress-Related Biomarkers Domain

The epigenetic domain and stress-axis biomarkers reflect the dynamic dimension of biological regulatory burden associated with chronic stress, trauma, and exposure to psychoactive substances. Epigenetic modifications influence synaptic plasticity and the consolidation of compulsive patterns, thereby modulating the stability of the Regulatory Control State [28,29].

Within the MAC/MAB–RCS framework, these indicators are interpreted as contextual signals of regulatory pressure that may precede or accompany clinically observable deterioration in control. Their role is limited to supporting clinical reasoning and validation research, without serving as standalone diagnostic or prognostic markers.

### 4.4. Psychological Phenotype Domain: Clinical Expression of RCS

The psychological phenotype constitutes the direct clinical expression of Regulatory Control State functioning. Traits such as impulsivity, reward sensitivity, stress tolerance, and emotional regulation strategies shape how biological vulnerability manifests behaviorally [30,31].

In the MAC/MAB–RCS model, these parameters are treated as dynamic and potentially modifiable components of RCS rather than as relatively fixed personality traits. Their modification represents a key target of MAB-oriented interventions, aimed at restoring functional regulatory control and reducing compulsivity. Importantly, within the MAC/MAB–RCS framework, psychological features are interpreted in terms of their momentary contribution to regulatory capacity, rather than as stable diagnostic or personality dimensions.

### 4.5. Neurobiological Network Domain

The neurobiological domain in the MAC/MAB–RCS model focuses on large-scale neural network functioning rather than isolated brain structures. Of particular importance is the balance between executive control networks, reward-related circuits, and the salience network, whose dysregulation promotes the dominance of reactivity over control and the escalation of compulsive behaviors [32,33,34].

A key insight from the literature is the partial reversibility of these alterations. Normalization of regulatory network functioning correlates with improved Regulatory Control State stability and reduced relapse risk, providing neurobiological support for the effectiveness of psychotherapeutic, behavioral, and pharmacological interventions [35]. Notably, RCS is not equated with any single neural network configuration, but reflects the functional balance among interacting regulatory systems.

### 4.6. Integration Across Domains: From Data to Clinical Interpretation

A defining feature of the MAC/MAB–RCS model is its approach to multidomain data integration. Genetic, epigenetic, neurobiological, and psychological information is neither summed nor algorithmically weighted but interpreted through its influence on the stability of the Regulatory Control State.

This approach enables the construction of a functional regulatory profile that supports decisions regarding monitoring intensity, the proportional balance between MAC and MAB strategies, and the timing of intervention escalation or de-escalation [36,37]. This interpretative integration preserves clinical judgment and avoids black-box decision-making, positioning RCS as a clinician-facing construct rather than an automated risk score.

## 5. Discussion

### 5.1. Bridging Neurobiological Knowledge and Clinical Decision-Making in Addiction Psychiatry

The aim of this work was to propose an integrative conceptual framework addressing the persistent gap between the rapidly expanding neurobiological knowledge of addiction disorders and its practical application in everyday clinical decision-making [1,2,3,4,5]. Although contemporary addiction research has generated detailed models of neural circuitry, stress reactivity, and reward dysregulation, these advances have translated only partially into tools that directly inform when, how intensively, and in what manner interventions should be deployed in routine clinical care [6,7,10,11].

Prevailing diagnostic systems and theoretical frameworks provide essential descriptive and explanatory value but remain limited in their ability to capture the dynamic, state-dependent nature of relapse vulnerability. Diagnostic categories typically reflect cumulative symptom burden rather than momentary regulatory instability, while domain-based research frameworks often fragment clinically relevant information across parallel dimensions without offering a unifying parameter for real-time therapeutic decision-making. As a result, clinicians are frequently required to integrate heterogeneous biological, psychological, and contextual signals intuitively, without a shared conceptual scaffold that links these signals to proportional intervention strategies.

The MAC/MAB–RCS model was developed in direct response to this translational challenge. Rather than introducing a competing theory of addiction, the framework offers a functional synthesis that organizes multidomain information around a single, clinically interpretable question: the individual’s current capacity for self-regulation. By conceptualizing addiction-related vulnerability in terms of the stability of the Regulatory Control State (RCS), the model provides a state-sensitive reference point that complements existing neurobiological and transdiagnostic approaches.

In this sense, the MAC/MAB–RCS framework is explicitly positioned as interoperable with established models such as the Research Domain Criteria (RDoC), the Addictions Neuroclinical Assessment (ANA), and contemporary addiction neurocircuitry theories. Whereas RDoC structures psychopathology into functional domains and ANA systematizes multidomain assessment, the MAC/MAB–RCS model introduces an integrative, state-level construct designed to support time-sensitive clinical decisions regarding intervention sequencing, proportionality, and escalation [8,10,11,12,13]. Importantly, the framework does not reduce clinical complexity to a single biomarker or algorithmic score but preserves the interpretative role of clinical judgment.

From a translational perspective, the Regulatory Control State offers a conceptual bridge between mechanistic research and clinical practice. RCS enables the interpretation of fluctuating signals—such as craving variability, stress load, executive control lapses, and contextual risk exposure—as manifestations of a common regulatory process, rather than as isolated symptoms or parallel risk factors. This reframing supports a shift from static relapse prediction toward dynamic risk trajectory monitoring, in which intervention strategies are adjusted in response to emerging regulatory destabilization.

These conceptual foundations also open avenues for future applications of the MAC/MAB–RCS model in patient-monitoring and decision-support tools. In particular, the framework is compatible with ecological momentary assessment (EMA) approaches, allowing repeated, low-burden sampling of state-level indicators relevant to RCS stability across contexts and time. In this formulation, RCS may function as an integrative state parameter that can interface with digital health technologies and emerging artificial intelligence–assisted systems, while avoiding reliance on rigid predictive algorithms or black-box decision-making.

Taken together, the MAC/MAB–RCS model addresses a critical unmet need in addiction psychiatry by providing a clinically oriented, theoretically grounded framework that translates complex neurobiological and psychological knowledge into actionable principles for individualized, dynamic care.

### 5.2. Regulatory Control State as a Functional Clinical Construct

The central contribution of the MAC/MAB–RCS model is the introduction of the Regulatory Control State (RCS), as defined in Section 3.2, a higher-order functional clinical construct. RCS organizes heterogeneous diagnostic information—including biological vulnerability, neural network functioning, and psychological phenotype—into a coherent, dynamic indicator of the individual’s current self-regulatory capacity [8,12,13,14,15]. RCS is conceptualized as a dynamic indicator of regulatory stability that supports state-sensitive clinical interpretation and intervention planning.

In practical terms, the Regulatory Control State differs from integrative research frameworks such as the Research Domain Criteria (RDoC) and the Addictions Neuroclinical Assessment (ANA) by its role in real-time clinical decision-making rather than multidimensional assessment architecture. While RDoC and ANA organize clinical and biological information across parallel domains to support comprehensive characterization, RCS functions as a unifying, state-level reference point that directly informs when to prioritize stabilization-oriented versus behavior-modulation–oriented interventions.

Thus, RCS is not intended to replace domain-based assessment but to translate its outputs into a single, clinically interpretable indicator of current regulatory stability, enabling time-sensitive decisions regarding intervention sequencing, proportionality, and escalation without requiring repeated full-domain reassessment.

This perspective shifts emphasis from categorical diagnosis to assessment of regulatory state, aligning with the principles of precision psychiatry and risk-stratification–based approaches [6,7,14]. RCS does not define enduring traits or clinical identity but describes a variable level of regulatory system burden that can change in response to therapeutic interventions and environmental factors. Importantly, conceptualizing regulation as a state rather than a trait emphasizes its potential reversibility and responsiveness to timely clinical intervention.

In this sense, RCS functions as an integrative construct that enables functional interpretation of multilevel data without reducing clinical complexity to isolated biological or behavioral markers.

### 5.3. Clinical Implications: From Reactive Treatment to Risk Trajectory Management

Clinically, the MAC/MAB–RCS model supports a shift away from purely reactive treatment of fully developed relapse episodes toward earlier identification of escalating risk [13,16,17]. Destabilization of the Regulatory Control State often precedes overt loss-of-control episodes, making it a useful reference point for intervention planning and relapse risk trajectory management.

The distinction between Minimizing Addiction Craving (MAC) and Modulation of Addictive Behaviors (MAB) strategies allows flexible tailoring of therapeutic actions to the patient’s current regulatory profile. High craving burden and stress exposure favor stabilization-focused interventions, whereas relative RCS stability justifies the implementation of strategies aimed at modifying behavioral patterns and strengthening executive control.

Although the MAC/MAB–RCS framework does not prescribe fixed thresholds or algorithmic rules for shifting between intervention strategies, proportional transitions can be standardized at the level of decision logic rather than numerical scoring. Specifically, shifts between MAC- and MAB-dominant approaches are anchored to predefined clinical patterns, such as sustained convergence of state-level indicators, loss or recovery of executive control, and persistence of behavioral spillover across contexts. Systematic documentation of these patterns over time allows clinical decisions to remain transparent, reviewable, and auditable, even in the absence of formalized metrics.

In this sense, consistency across clinicians is supported through shared interpretative criteria and explicit decision rationale, rather than rigid cutoffs. This approach aligns with stepped-care and risk-stratification models in psychiatry, where proportionality and clinical judgment are preserved while maintaining accountability and reproducibility of treatment decisions.

### 5.4. System-Level Implications and Compatibility with Stepped Care Models

At the system level, the MAC/MAB–RCS model is compatible with stepped care architectures widely used in psychiatry and addiction treatment across Europe and the United States. These approaches emphasize matching intervention intensity to current clinical need while optimizing use of available therapeutic resources [16,29].

Application of the MAC/MAB–RCS framework does not require comprehensive biological diagnostics at every stage of care but is grounded in principles of proportionality and adaptability. The model may support earlier identification of patients at risk of regulatory destabilization and reduce costs associated with relapse treatment, while remaining consistent with chronic disease management principles and value-based care approaches [16,29,36]. This flexibility supports incremental implementation across different levels of care, from outpatient settings to specialized treatment programs.

### 5.5. Limitations and Responsible Interpretation of the Framework

The MAC/MAB–RCS model constitutes a conceptual framework requiring further empirical validation. As defined in Section 3.2, the Regulatory Control State (RCS) is a functional construct for which no single direct measurement indicator currently exists, limiting its immediate operationalization in quantitative research and multicenter comparisons [24,36]. Accordingly, the framework should not be interpreted as a diagnostic algorithm or deterministic predictive tool, but as a structured aid for clinical reasoning and hypothesis generation.

Importantly, the framework explicitly accounts for heterogeneity across different types of addiction, including substance-related and behavioral addictions. Within the MAC/MAB–RCS framework, RCS is not associated with fixed or universal destabilization thresholds. Instead, regulatory destabilization is defined in relative and functional terms, based on within-individual changes in recovery capacity, cross-domain interference, and behavioral control over time. While the phenomenological expression of craving, stress reactivity, or compulsive behavior may differ across addiction types, the underlying logic of regulatory stability and destabilization remains consistent. This approach allows the framework to accommodate heterogeneity in clinical presentation without assuming uniform cutoff values across disorders.

A potential conceptual risk of the MAC/MAB–RCS framework is circular reasoning when craving, stress, or executive control are considered both as inputs to RCS estimation and as outcomes of regulatory destabilization. The present framework addresses this risk by emphasizing temporal ordering, functional dissociation, and trajectory-based interpretation. State-level indicators are used to estimate RCS prospectively, based on their convergence and persistence over time, whereas RCS destabilization is inferred from subsequent loss of recovery capacity, cross-domain interference, and behavioral spillover. In this formulation, the same variables may appear at different stages of clinical reasoning but serve distinct inferential roles, reducing the risk of tautological interpretation.

An additional limitation concerns the current availability and clinical feasibility of biological and neurobiological biomarkers relevant to addiction-related regulatory processes. Many candidate biomarkers (e.g., stress-axis measures, epigenetic markers, or neuroimaging-derived network parameters) remain costly, context-dependent, and unevenly accessible across clinical settings. As a result, their routine use in everyday addiction care is limited, particularly outside specialized or research-oriented centers.

For this reason, the MAC/MAB–RCS framework does not assume the availability of biomarkers as a prerequisite for clinical application. Instead, biological indicators are conceptualized as optional, contextual sources of information that may refine vulnerability stratification or monitoring intensity when available, but are not required for initial RCS estimation or intervention selection. This design choice reflects a deliberate emphasis on proportionality, clinical feasibility, and equity of application across diverse healthcare environments.

Although the present framework is grounded in a synthesis of the existing literature, empirical validation remains a key priority for future research. Initial predictive testing could be conducted in prospective cohort designs, in which RCS is operationalized as a composite, state-sensitive construct integrating biological markers (e.g., cortisol indices and FKBP5-related measures), neuroimaging-derived network parameters (e.g., rs-fMRI connectivity within and between the default mode and salience networks), and psychological assessments (e.g., BIS/BAS indices of impulsivity and approach motivation). Such a multimodal, transdiagnostic measurement logic is conceptually consistent with frameworks like the Addictions Neuroclinical Assessment (ANA), which emphasize the integration of neurobiological and clinical dimensions to improve clinical characterization and risk stratification [38]. Importantly, the marked heterogeneity of substance use disorders (e.g., differences between opioid-related and behavioral addictions) should be addressed through subgroup stratification and sensitivity analyses, to reduce oversimplification and to better reflect individual risk trajectories. From a validation perspective, three complementary study designs appear particularly informative. First, longitudinal cohort studies with repeated state-level assessments (e.g., EMA-based designs) can test whether changes in RCS precede relapse or recovery trajectories. Second, stepped-care or adaptive intervention trials can evaluate whether RCS-guided switching between MAC- and MAB-dominant strategies improves clinical outcomes compared to standard care. Third, intervention studies targeting specific regulatory components (e.g., stress reactivity or executive control) can assess whether experimentally induced changes in these components translate into measurable shifts in RCS and downstream clinical risk.

Beyond measurement and validation challenges, important conceptual limitations also arise from the dynamic and context-dependent nature of regulatory processes. The RCS model assumes the integration of multiple domains into a dynamic regulatory state; however, interactions among these domains are inherently non-linear and context-dependent, as demonstrated by epigenetic research [29]. For example, histone modifications and DNA methylation patterns in stress-related genes (e.g., NR3C1) may be moderated by environmental exposures, introducing substantial heterogeneity into RCS-related predictions [29]. Moreover, biases in genetic data—such as population stratification in genome-wide association studies—may lead to spurious associations, particularly in clinically and ethnically diverse populations [26]. To address these challenges, future applications of the RCS framework may benefit from carefully applied advanced analytical approaches capable of modeling complex, non-linear interactions (e.g., Bayesian network–based or other machine learning methods), while remaining grounded in transparent, hypothesis-driven interpretation. Equally important are ethical considerations, including the avoidance of stigma or deterministic labeling based on biomarker-derived risk indicators. Accordingly, the MAC/MAB–RCS framework should not be interpreted as a deterministic predictive tool, but as an open conceptual model requiring critical contextual interpretation, consistent with ethical principles of precision psychiatry and aimed at minimizing risks of oversimplification, stigma, or deterministic labeling [27,28].

## 6. Conclusions

This work presents the MAC/MAB–RCS model as an integrated conceptual framework designed to support risk stratification, early identification of regulatory destabilization, and clinically informed personalization of interventions in addiction psychiatry. At the core of the model lies the construct of the Regulatory Control State (RCS), conceptualized as a dynamic, functional indicator of an individual’s capacity for self-regulation in the context of biological, psychological, and environmental burdens.

The model does not seek to replace existing neurobiological theories or diagnostic systems such as the DSM or ICD. Instead, it provides a translational framework for clinically meaningful interpretation of multilevel data. By integrating information on biological vulnerability, neural network functioning, and psychological phenotype, the MAC/MAB–RCS framework supports a shift from reactive treatment toward proactive management of relapse risk trajectories.

The distinction between Minimizing Addiction Craving (MAC) and Modulation of Addictive Behaviors (MAB) strategies allows for flexible tailoring of interventions to the patient’s current regulatory profile, without reducing clinical complexity to a single diagnostic parameter. Although the model requires further empirical validation, it offers a useful and testable framework for future research and the development of clinical practice in addiction psychiatry. Future empirical studies should focus on the operationalization of RCS and the evaluation of its utility in longitudinal and interventional research designs. Importantly, the MAC/MAB–RCS framework is intended as a heuristic scaffold for clinical reasoning rather than as a prescriptive algorithm.

## Data Availability

Not applicable. No new data were created or analyzed in this study.
